# Dynamic mechanisms of neuroligin-dependent presynaptic terminal assembly in living cortical neurons

**DOI:** 10.1186/1749-8104-9-13

**Published:** 2014-05-29

**Authors:** Luke AD Bury, Shasta L Sabo

**Affiliations:** 1 Department of Pharmacology, Case Western Reserve University School of Medicine, Cleveland, OH 44106, USA; 2 Neuroscience, Case Western Reserve University School of Medicine, Cleveland, OH 44106, USA

**Keywords:** Synaptogenesis, Axonal transport, Neuroligin, Neurexin, Syncam, Presynaptic, Trans-synaptic adhesion

## Abstract

**Background:**

Synapse formation occurs when synaptogenic signals trigger coordinated development of pre and postsynaptic structures. One of the best-characterized synaptogenic signals is trans-synaptic adhesion. However, it remains unclear how synaptic proteins are recruited to sites of adhesion. In particular, it is unknown whether synaptogenic signals attract synaptic vesicle (SV) and active zone (AZ) proteins to nascent synapses or instead predominantly function to create sites that are capable of forming synapses. It is also unclear how labile synaptic proteins are at developing synapses after their initial recruitment. To address these issues, we used long-term, live confocal imaging of presynaptic terminal formation in cultured cortical neurons after contact with the synaptogenic postsynaptic adhesion proteins neuroligin-1 or SynCAM-1.

**Results:**

Surprisingly, we find that trans-synaptic adhesion does not attract SV or AZ proteins nor alter their transport. In addition, although neurexin (the presynaptic partner of neuroligin) typically accumulates over the entire region of contact between axons and neuroligin-1-expressing cells, SV proteins selectively assemble at spots of enhanced neurexin clustering. The arrival and maintenance of SV proteins at these sites is highly variable over the course of minutes to hours, and this variability correlates with neurexin levels at individual synapses.

**Conclusions:**

Together, our data support a model of synaptogenesis where presynaptic proteins are trapped at specific axonal sites, where they are stabilized by trans-synaptic adhesion signaling.

## Background

Upon axon-dendrite contact, the extracellular domains of axonal and dendritic adhesion proteins interact, leading to recruitment of synaptic proteins and subsequent synapse formation [1-3]. A variety of synaptogenic adhesion partners have been discovered, including neurexin/neuroligin, SynCAM/SynCAM, and neurexin/LRRTM2, among others [4-15]. Of these, the most studied trans-synaptic pair is postsynaptic neuroligin and its interaction with presynaptic neurexin.

Although a number of studies have focused on understanding the functions and mechanisms of trans-synaptic adhesion molecules in recent years, it remains unclear how synaptogenic adhesion results in presynaptic protein recruitment and synaptogenesis [16,17]. Importantly, it is not known whether trans-synaptic adhesion actively attracts synaptic proteins to sites of signaling or acts primarily to stabilize proteins at nascent terminals. In addition, it is unknown whether synaptic protein recruitment proceeds continuously until the site is saturated, or if SV and AZ protein levels are modulated even at the earliest stages of development, as they are at mature synapses [18-25]. Finally, it is unclear if trans-synaptic adhesion regulates synaptic protein levels at individual synapses or primarily creates sites in the axon that are capable of synapse formation while other cellular processes regulate levels of recruitment.

To answer these questions, we induced synaptogenesis via contact with neuroligin-1 and SynCAM-1 then employed short- and long-term time-lapse confocal imaging of presynaptic protein recruitment to nascent sites of trans-synaptic signaling. This paradigm allowed us to record recruitment from the initiation of trans-synaptic adhesion onward. We found that SV protein recruitment to individual sites of trans-synaptic adhesion fluctuated, as did protein recruitment at axon-dendrite synapses. These changes in synaptic protein levels strongly correlated with the amount of neurexin-1β at individual synapses. However, trans-synaptic adhesion did not attract SV or AZ proteins to contacts or significantly alter synaptic protein transport. Unexpectedly, neurexin alone was not the primary signal for recruitment since synaptic proteins were specifically recruited to sites of enhanced neurexin-1β clustering, even though neurexin was present throughout the contact region. Overall, our data support a model of synaptogenesis in which presynaptic proteins are trapped at, but not actively attracted to, specific axonal sites, where they are stabilized by trans-synaptic adhesion signaling.

## Results

### Synaptic protein recruitment fluctuates frequently and rapidly during assembly of individual presynaptic terminals

**Figure 1:**
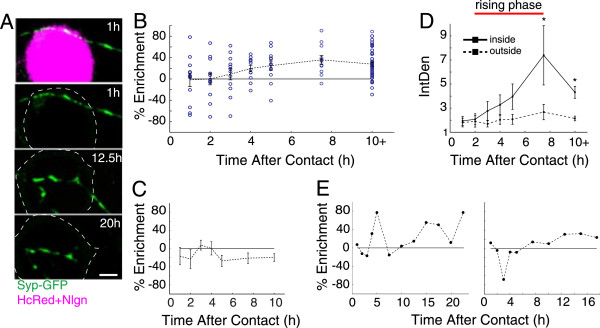
**Levels of synaptic vesicle protein enrichment at individual trans-synaptic adhesion sites are modulated throughout recruitment. (A)** Images from live, long-term, time-lapse confocal imaging of an axon expressing synaptophysin-GFP (*green)* and contacting an HEK293 cell (*magenta* and *white outline*) that expresses neuroligin-1 + HcRed. The top panel shows an axon and HEK293 cell at one hour after contact. In this panel, synaptophysin-GFP at the contact site appears *white* in the overlay. The remaining panels show only synaptophysin fluorescence for clarity. Images were collected 1 hour, 12.5 hours and 20 hours after contact was induced (scale bar = 5 μm). **(B)** Time course of STV enrichment in axonal regions that contact neuroligin-1-expressing cells. Enrichment corresponds to the difference between the total STV integrated density within the contact region and outside of the contact, normalized to the total STV integrated density throughout the axon and expressed as a percentage. Positive values indicate enrichment inside the contact area. Individual points (*cyan*) represent mean values from 45 images collected at 10 second intervals, beginning at the time indicated on the x-axis. *Dashed line,* overall mean at each time point (n = 12 contacts). Overall, enrichment increased gradually over the first ten hours then remained elevated. Error bars = SEM. **(C)** Over the same time course, STVs were not enriched at sites of contact between axons and HEK293 cells expressing HcRed but no neuroligin. **(D)** Integrated density of synaptophysin-GFP inside (*solid line*) and outside (*dashed line*) of contacts over time. *Red bar,* rising phase of enrichment (2 to 7.5 hours after contact). Synaptophysin increased at contacts while remaining stable outside contacts. **(E)** Enrichment for two axons. At individual contacts, enrichment was highly dynamic throughout the imaging period. Therefore, although STV enrichment increases at trans-synaptic adhesion sites overall, enrichment levels for individual axons vary throughout the first 24 hours of development.

Contact between an axon and dendrite is one of the first steps of synapse formation; however, it is difficult to predict when and where axo-dendritic contact will occur and whether synapses will form at these sites. To overcome this challenge, we developed a strategy for long-term imaging of synaptic protein recruitment to nascent sites of presynaptic terminal formation, initiated by contact with neuroligin-1-expressing HEK293 cells as a proxy for postsynaptic dendrites. Specifically, 7 to 10 days *in vitro* (DIV) cortical neurons were sparsely transfected with synaptophysin-GFP, to label synaptic vesicle protein transport vesicles (STVs), which deliver SV proteins to developing presynaptic terminals [7,26-43]. We then imaged STVs in individual axons that contacted neuroligin-1-expressing HEK293 cells for up to 25 hours after contact (Figure 1A, Additional file 1), collecting multiple ‘short sequences’ of rapid imaging (every 10 seconds for 7.5 minutes) separated by 1 to 2.5 hours. The ‘short sequences’ granted the ability to track rapid STV movements, observe recruitment of individual STVs, and distinguish between STVs that were stably recruited and those that were *en route* through the contact site while preserving neuron health. With this approach, it was possible to resolve a full time-course of presynaptic protein recruitment at individual sites of trans-synaptic signaling in individual axons.

**Figure 2:**
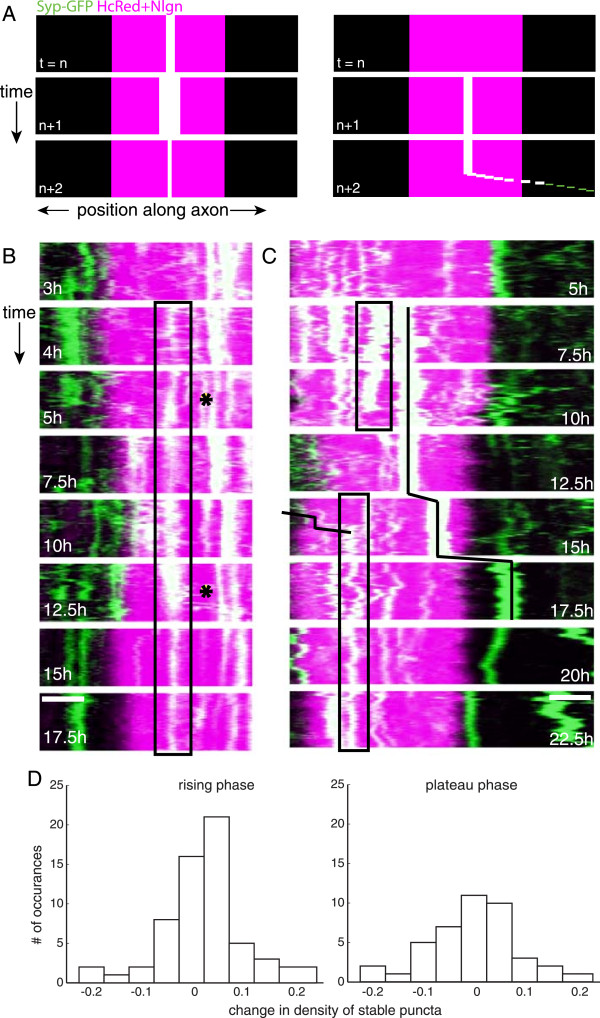
**Modulation of synaptic vesicle protein recruitment occurs through two distinct mechanisms. (A)** Model kymographs illustrating that variability in protein levels could arise from fluctuations in synaptic protein levels at individual recruitment sites (*left*) or through the addition/subtraction of entire recruitment sites (*right*). **(B, C)** Kymographs of STVs (*green*) in axons that contact neuroligin-1-expressing HEK-293 cells (*magenta*). (B, *box*) Synaptophysin-GFP levels at stable contacts are variable over the course of hours and even minutes (*asterisks*). (C, *top box, top line*) Sites of recruitment that are stable over the course of hours can be eliminated, sometimes rapidly. (C) To replace these sites, individual STVs can be captured (*bottom line*) and stabilized (*bottom box*) at contact sites. t = time after contact, scale bars = 5 μm. **(D)** Histograms of net changes in the densities of individual stable recruitment sites from one short imaging sequence to the next. Stable sites were defined as puncta that appeared at a given site throughout at least one short imaging sequence. The number of stable sites of recruitment varied at the majority of contacts during both the rising and plateau phases, with a bias toward puncta addition during the rising phase (*left*) but not the plateau phase (*right*).

To quantify STV recruitment within individual axons, the integrated densities of all STVs were summed in each axonal region that made contact with a neuroligin-1-expressing HEK293 cell and compared to regions without contact in the same axon. Since integrated density corresponds to the sum of pixel intensities, STVs with higher integrated densities were brighter and/or larger, indicating more SV protein. Enrichment was calculated by determining the difference between fluorescence (integrated density per length of axon) inside and outside the contact region, divided by the sum of the fluorescence inside and outside. Overall, in regions that displayed recruitment (see Methods), synaptophysin-GFP was increasingly enriched at sites of trans-synaptic adhesion over the first ten hours of imaging (Figure 1B; n = 12 contacts), similar to previous observations using immunocytochemistry to examine populations of axons [40]. No enrichment was observed over the same time course when axons were contacted with HEK293 cells expressing only HcRed, with no neuroligin, indicating that the observed recruitment was a specific result of trans-synaptic adhesion (Figure 1C; n = 5 contacts). Enrichment at neuroligin contacts was due to increased SV protein recruitment to contacts without substantial changes in SV protein outside areas of contact (Figure 1D). Within contact areas, SV protein levels started to rise two hours after contact was initiated. This ‘rising/recruitment phase’ progressed until reaching a plateau ten hours after contact initiation (Figure 1D). However, recruitment at individual contacts was quite labile (Figure 1E). Throughout the imaging period, absolute levels of recruited synaptophysin-GFP increased or decreased from one hour to the next, even for axons that displayed enrichment for most or all of the 24 hour imaging period. These substantial fluctuations occurred during both rising and plateau phases. Importantly, variability in fluorescence was not caused by focal drift, since we employed Perfect Focus correction to maintain the focus (see Methods). Therefore, in individual axons, recruitment of proteins does not consistently increase: rather, levels of recruited SV proteins fluctuate while remaining elevated compared to neighboring axonal areas without trans-synaptic signaling.Fluctuations in recruited SV protein levels could occur through at least two mechanisms (Figure 2A). Once formed, sites of STV recruitment could be stable while the level of protein at each stable site fluctuates. Alternatively, the sites themselves could form and disappear. These two mechanisms are not mutually exclusive, and our live imaging revealed both types of variability. Synaptophysin-GFP levels fluctuated at individual sites that were stable over several hours (Figure 2B). Fluctuations often occurred very rapidly, within minutes. In addition, sites of stable recruitment often persisted for multiple hours before completely, and sometimes immediately, disbanding (Figure 2C). Instant formation of stable sites was also observed (Figure 2C).

To assess the prevalence of fluctuations in synaptophysin recruitment levels at individual sites, we determined the number of sites of stable STV accumulation that displayed fluctuations in synaptophysin-GFP levels over time. Stable accumulation sites were defined as sites in the axon where synaptophysin-GFP signal was consistently present over the course of at least one short imaging sequence. At 24.9% of individual stable accumulation sites (n = 115), there were observable fluctuations of synaptophysin-GFP within short imaging sequences. For stable accumulation sites that persisted over multiple short imaging sequences, synaptophysin-GFP levels changed between short imaging sequences 18.4% of the time (n = 103). It should be noted that these are most likely conservative estimates of the actual synaptic vesicle protein level fluctuations at stable sites, as small fluctuations of protein at these sites are difficult to observe.Additions and losses of entire stable sites of recruitment resulted in net changes in the density of discrete, stable recruitment sites from one short sequence to the next (that is over 1 to 2.5 hours) in 76.6% of imaging sequences during the rising phase and 73.8% during the plateau phase. This indicates that sites of recruitment were highly labile throughout synapse assembly. During the rising phase of recruitment, the additions and losses resulted in net increases in the density of stable sites of recruitment in 54.7% of imaging sequences (Figure 2D; n = 64). In contrast, during the plateau phase, appearances and disappearances were more balanced, with net increases in discrete contact sites only 38.1% of the time (Figure 2D).

### Levels of synaptic vesicle proteins are highly labile at developing axo-dendritic contacts

**Figure 3:**
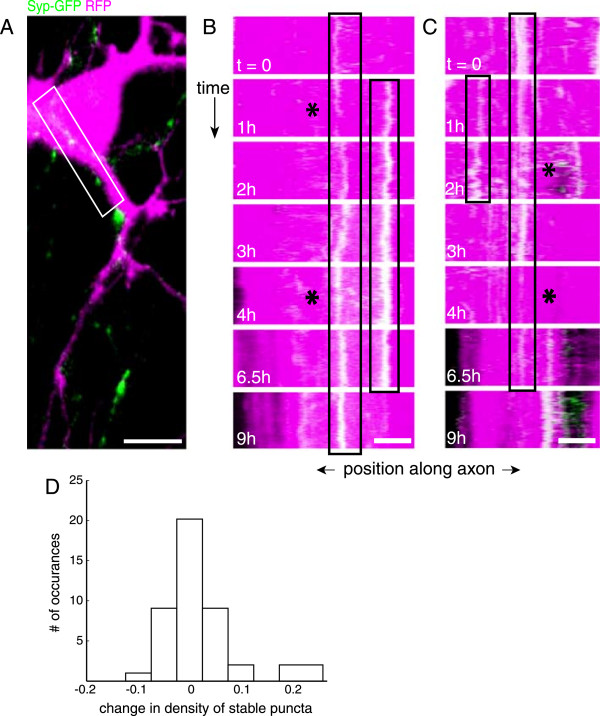
**Synaptic vesicle protein recruitment to axo-dendritic contacts is similar that at induced trans-synaptic signaling sites. (A)** Image of an axon transfected with synaptophysin-GFP (*green*) contacting somato-dendritic region from an adjacent cell transfected with RFP (*magenta*). *White box* corresponds to contact region represented by kymograph in (B). **(B, C)** Kymographs of STVs (*green*) in axons that contact RFP-expressing dendrites from different neurons (*red*). *Boxes*, as seen in induced axo-dendritic contacts, synaptophysin-GFP levels are variable at individual stable sites in neuron-neuron contacts over the course of hours and minutes (*asterisks*). t = time after imaging begins; scale bars (A) = 10 μm, (B, C) = 5 μm. **(D)** Histogram of net changes in the densities of individual stable recruitment sites from one short imaging sequence to the next for neuron-neuron contacts. The number of stable sites of recruitment fluctuated between the majority of imaging sequences (corresponds to all non-zero bins), with a small bias toward addition of stable sites of recruitment.

To determine if the same types of fluctuations in SV protein recruitment occur at neuron-neuron synapses, we imaged regions of contact between axons expressing synaptophysin-GFP and morphologically identified dendrites and somas expressing RFP. Contacts were imaged for 12 to 24 hours at 7 to 10 DIV with the imaging protocol described above (see Additional file 2, Figure 3A). Axo-dendritic and axo-somatic synapses exhibited many of the same dynamics observed in axons contacting neuroligin-1-expressing HEK293 cells. Levels of synaptophysin-GFP at individual sites of stable recruitment varied over time (Figure 3B, C). Synaptophysin-GFP protein levels at contact sites fluctuated 10.9% of the time (n = 43) between short imaging sequences (over 1 to 2.5 hours) and 17.6% of the time (n = 49) within short sequences (over 7.5 minutes). Furthermore, individual recruitment sites were established and dissolved over the course of hours (Figure 3B, C), with net changes in the density of stable sites of recruitment occurring in 55.5% of imaging sequences (Figure 3D; 31.1% additions and 24.4% losses; n = 45). These data indicate that the dynamics of synaptic vesicle protein recruitment at axo-dendritic synapses and hemi-synapses occurred over a similar time course. The similarities between recruitment dynamics at spontaneous neuron-neuron synapses and neuroligin-1-induced sites are especially striking since sites of recruitment may have been more mature in some neuron-neuron synapses, and synapse formation between neurons may involve additional signaling pathways. Together, the above data suggest that developing synapses undergo extensive fluctuations in presynaptic protein levels, even during initial formation.

### Synaptophysin is preferentially stabilized at clustered neurexin

**Figure 4:**
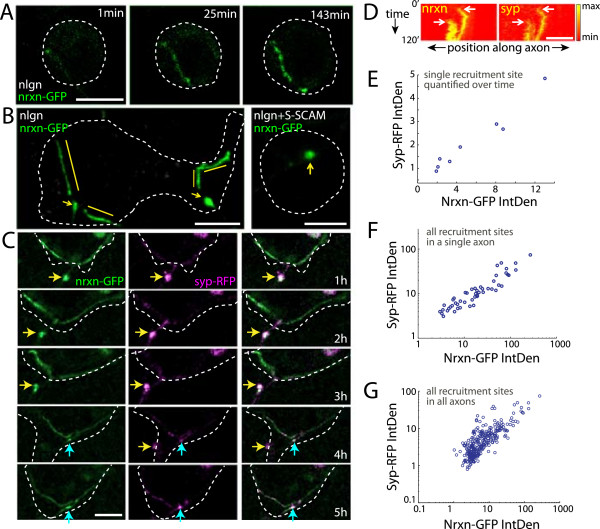
**Synaptic vesicle protein accumulation occurs at clustered neurexin and correlates with levels of clustered neurexin. (A)** Time-lapse images of an axon transfected with neurexin-1β-GFP (*green*) contacting a neuroligin-1-expressing HEK293 cell (*white outline*). Neurexin is recruited to contact sites within minutes and enhanced at these sites as imaging progresses. **(B)** Images of two axons that are transfected with neurexin-1β-GFP (*green*) and contact cells expressing neuroligin-1 (*left*, *white outline*) or neuroligin-1 + S-SCAM (*right, white outline*). At neuroligin-1-only contacts, neurexin appears both punctate (*arrows*) and diffuse (*bars*). Addition of S-SCAM enhanced neurexin-1β-GFP clustering and reduced diffuse neurexin-1β-GFP labeling at contacts (*arrow*). **(C)** Persistent sites of synaptophysin-RFP (*magenta*) recruitment appeared at sites of punctate neurexin-1β-GFP (*green*) recruitment (*arrows*). t = time after contact established. **(D)** Kymographs of neurexin-1β-GFP and synaptophysin-RFP, starting immediately after contact with a neuroligin-1-expressing cell was established. Neurexin and synaptophysin clustered simultaneously at the same sites in the axon (*arrows*). **(E)** At an individual recruitment site, synaptophysin-RFP and neurexin-1β-GFP levels were positively (r = 0.98) and significantly (*P* = 2.0 × 10^−5^) correlated over several hours of imaging. This correlation was significant for 11 of 14 co-recruitment sites that were present for at least 5 hours. **(F)** When combining data from all recruitment sites in the same axon, synaptophysin and neurexin integrated densities were also correlated (r = 0.91, *P* = 6.0 × 10^−20^). Similar correlation was observed in five of seven axons. **(G)** When the data from all axons were pooled, neurexin and synaptophysin were positively and significantly correlated (*P* = 1.0 × 10^−74^, r = 0.82). (A-C) scale bars = 10 μm, (D) scale bar = 5 μm, (E-G) correlations made from non-transformed data and plotted in log-log form for display purposes.

Neuroligin induces synaptogenesis through its presynaptic binding partner neurexin [1,44]. To ascertain the time course of neurexin clustering at contacts, neuroligin-1-expressing HEK293 cells were added to neuronal cultures transfected with neurexin-1β-GFP. Then contacts between neuroligin-1-expressing HEK293 cells and axons expressing neurexin-1β-GFP were imaged immediately. Prior to contact, neurexin-1β-GFP appeared dim and diffuse within the axon. Upon contact with neuroligin-1-expressing HEK293 cells, neurexin-1β-GFP began to be recruited to sites of contact within minutes, and showed strong recruitment within 1 to 2 hours (Figure 4A-C, n = 14 contacts), consistent with previous data [40]. Because we have not found any neurexin-β selective antibodies for immunofluorescence, we could not determine the extent to which neurexin-1β-GFP was over-expressed in these experiments. However, similar fluorescently tagged neurexin-1β-GFP has been used in a number of studies to investigate the localization, trafficking and function of neurexin [45-48]. Importantly, the distribution pattern of α-neurexin is not significantly influenced by the level of neurexin expressed [48]. Therefore, although over-expression of neurexin in neurons increases synapse density [46], it not expected to change the dynamics or distribution of neurexin.

Neuroligin-1 recruited neurexin-1β-GFP to the entire contact region, where neurexin appeared in both punctate and diffuse forms, even within the same contacts (Figure 4B). Recent work has shown that the synaptogenic effect of neuroligin depends on the postsynaptic scaffolding protein S-SCAM, which works, in part, by clustering neuroligin [15]. To determine if postsynaptic clustering of neuroligin facilitates presynaptic clustering of neurexin, HEK293 cells that co-expressed S-SCAM and neuroligin-1 were dropped onto neurons that were transfected with neurexin-1β-GFP. This led to enhanced clustering of neurexin-1β-GFP in axons that contacted transfected HEK293 cells (Figure 4B), suggesting that the synaptogenic effects of S-SCAM-induced neuroligin clustering might be due to enhanced clustering of presynaptic neurexin. Indeed, artificial clustering of neurexin is sufficient to induce presynaptic protein recruitment [16], and recent work suggests that neurexin clustering is critical for the synaptogenic function of the neurexin-neuroligin interaction in *Drosophila*[49].This raises the question of whether synaptic proteins are preferentially recruited to punctate clusters of neurexin or whether they can be recruited to any site with neurexin enrichment. To test this, neurons were co-transfected with neurexin-1β-GFP and synaptophysin-RFP. Neuroligin-1-expressing HEK293 cells were then added to these cultures, and contacts were imaged for up to 24 hours, as described above. Although there was a combination of diffuse and punctate recruitment of neurexin-1β-GFP to contacts (Figure 4A-C), stable sites of synaptophysin-RFP recruitment were almost exclusively co-localized with neurexin-1β-GFP puncta (Figure 4C). Surprisingly, neurexin-1β-GFP clustering and synaptophysin-RFP clustering occurred nearly simultaneously at most shared sites (Figure 4D, n = 13/18 clusters). Neurexin clustered concurrently with SV protein recruitment even when HEK293 cells expressed both neuroligin-1 and S-SCAM to enhance neurexin clustering. These data support a model in which discrete neurexin clustering is critical for stable, rapid, neuroligin-induced presynaptic protein recruitment in mammalian cortical neurons.

### Levels of recruited synaptophysin and neurexin are strongly correlated at developing presynaptic terminals

Our observation that SV proteins and neurexin selectively and simultaneously co-cluster raises the question of whether synaptophysin recruitment and neurexin clustering are coordinated. To test this, we first determined whether synaptophysin and neurexin levels were correlated at individual sites of recruitment. Indeed, synaptophysin levels were positively and significantly correlated with neurexin levels at individual co-clusters that were present for at least five consecutive short sequences of imaging (Figure 4E, *P* < 0.05 at 11/14 sites from 7 axons, r = 0.37 to 0.98). Neurexin-1β-GFP and synaptophysin-RFP levels were also significantly correlated when all clusters from individual axons were pooled (Figure 4F, *P* < 0.05 in 5/7 axons, r = 0.52 to 0.96) or when all axons were combined (Figure 4G, n = 307 sites, *P* = 1.0 × 10^−74^, r = 0.82). Therefore, recruitment of SV proteins and clustered neurexin are coordinated during presynaptic terminal assembly.

**Figure 5:**
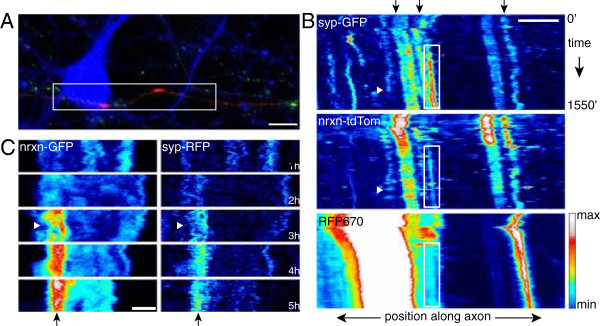
**Neurexin and synaptophysin levels fluctuate over the course of minutes at axo-dendritic contact sites and neuroligin induced developing synapses. (A)** Image of an axon co-expressing synaptophysin-GFP (*green*) and neurexin-tdTomato (*red*) and contacting the somato-dendritic region of a neuron expressing RFP670 (*blue*). *White box* corresponds to contact region represented by kymographs in (B). **(B)** Individual kymographs of an axon expressing synaptophysin-GFP (*top*) and neurexin-tdTomato (*middle*) that contacts a dendrite expressing RFP670 (*bottom*). Levels of both neurexin and synaptophysin fluctuate over the course of minutes to hours at sites that contact somato-dendritic regions of the adjacent neuron (*arrows*, *top*), including the formation (*white box*) and disappearance (*white arrowhead*) of co-clusters. **(C)** Kymographs of an axon expressing neurexin-GFP (*left*) and synaptophysin-RFP (*right*) contacting a neuroligin-expressing HEK293 cell. The amount of neurexin and synaptophysin at co-clustered sites (*arrow, bottom*) fluctuates extensively over hours and even within minutes (*white arrowheads*). Scale bars (A, B) = 10 μm, scale bar (C) = 5 μm.

Next, we asked whether the long-term dynamics of synaptophysin and neurexin recruitment were similar. To test this, 7 to 10 DIV neurons were transfected with either synaptophysin-GFP and neurexin-tdTomato or the near-infrared fluorescent protein RFP670 (Figure 5) [50]. Contacts between axons that expressed both synaptophysin-GFP and neurexin-tdTomato and dendrites that expressed RFP670 were then imaged over the course of 12 to 25 hours. Like synaptophysin, levels of neurexin fluctuated significantly over the course of long-term imaging at both spontaneously-formed axo-dendritic and axo-somatic neuron-neuron contacts (Figure 5A-B) as well as contacts formed with neuroligin-expressing HEK293 cells (Figure 5C). In addition, many neurexin clusters formed and disappeared over the course of hours (Figures 4C and 5), resembling the formation and dissolution of stable sites of synaptophysin recruitment (Figures 2 and 5).

### Trans-synaptic signaling recruits synaptic proteins without substantially altering their transport

**Figure 6:**
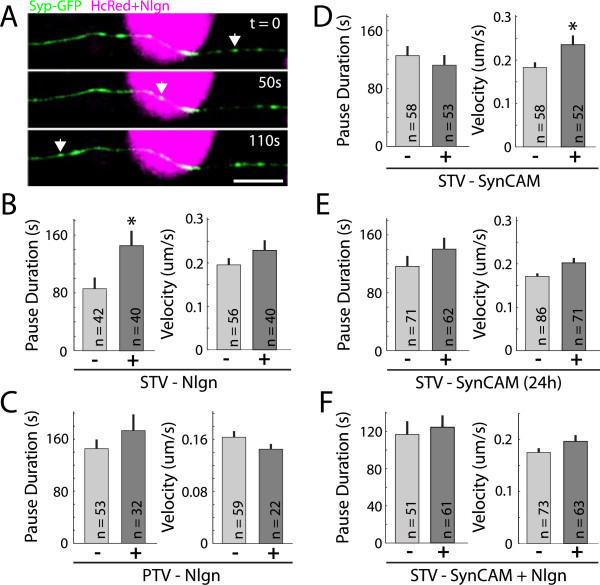
**Trans-synaptic signaling has little effect on synaptic vesicle or active zone protein transport. (A)** Time-lapse images of a neuroligin-1 + HcRed-expressing HEK293 cell (*magenta*) contacting an axon expressing synaptophysin-GFP (*green*). *Arrows*, position of individual STV in each frame. **(B)** Contact with neuroligin-1-expressing cells increases the mean pause duration but not instantaneous velocity of STVs inside the contact region. **(C)** For PTVs, neither the mean pause duration nor the instantaneous velocity was affected by neuroligin-1-induced trans-synaptic signaling. **(D)** Contact with SynCAM-1-expressing cells for one to four hours did not affect STV pause duration but increased their instantaneous velocity. **(E)** Contact with SynCAM-1 for 24 hours had no effect on STV pause duration or instantaneous velocity. **(F)** Contact with both neuroligin-1 and SynCAM-1 had no effect on STV pause duration or instantaneous velocity. *P < 0.05; t = time after imaging begins; error bars = SEM.

It is not known how STVs disengage from their microtubule tracks and become incorporated into synapses. Various signals that affect synaptogenesis also affect STV transport [38], and it has been suggested that neuroligin-neurexin signaling might recruit synaptic proteins by altering synaptic protein transport [51]. Therefore, we next tested the hypothesis that trans-synaptic signaling recruits presynaptic proteins by altering STV transport. To test this hypothesis, synaptophysin-GFP was imaged every 10 seconds for 7.5 minutes at 1 to 4 hours after contact was established between axons and neuroligin-1-expressing HEK293 cells (Figure 6A). Because STV transport is characterized by bursts of movement interrupted by pauses [27,29,31,33,34,38,41], we measured how fast STVs moved and how long they paused in the presence and absence of neuroligin-1. We found that STV pause duration increased at contacts with neuroligin-1-expressing cells when compared to contacts with HcRed-expressing control cells (Figure 6B; neuroligin = 145.3 ± 20.8 seconds, n = 40 pauses; no neuroligin = 85.7 ± 15.5 seconds, *P* = 0.0217, n = 42 pauses). However, there was no neuroligin-1-dependent change in instantaneous velocity (Figure 6B; neuroligin = 0.229 ± 0.023 μm/s, n = 40 movements; no neuroligin = 0.196 ± 0.015 μm/s, n = 56 movements, *P* = 0.391).

To investigate active zone protein transport, we looked at Piccolo-Bassoon transport vesicles (PTVs), which deliver active zone proteins to developing presynaptic terminals [30,39-41,52-59]. STVs and PTVs can be transported together in the axon [39,41,60]. Therefore, neuroligin-neurexin interactions could alter PTV transport, which in turn could recruit STVs. Similar to STVs, neuroligin-1 had no effect on the instantaneous velocity of PTVs (Figure 6C; neuroligin = 0.140 ± 0.008 μm/s, n = 22 movements; no neuroligin = 0.158 ± 0.009 μm/s, n = 59 movements, *P* = 0.473). Unlike STVs, contact with a neuroligin-1-expressing cell had no effect on the pause duration of PTVs (Figure 6C; neuroligin = 173.1 ± 24.9 seconds, n = 32 pauses; no neuroligin = 145.5 ± 14.0 seconds, n = 53 pauses, *P* = 0.634). Therefore, neuroligin-neurexin signaling does not recruit STVs indirectly via regulation of PTV transport.

To determine if other forms of synaptogenic trans-synaptic signaling alter STV movement, we also performed the live-imaging assay described above using HEK293 cells transfected with SynCAM-1, a trans-synaptic adhesion molecule that possesses synaptogenic properties similar to neuroligin-1 [13,14,61]. Unlike neuroligin-neurexin signaling, SynCAM-1-mediated trans-synaptic adhesion did not alter STV pause duration (Figure 6D; SynCAM = 112.5 ± 14.0 seconds, n = 53 pauses; no SynCAM = 125.5 ± 13.4 seconds, n = 58 pauses, *P* = 0.332). However, SynCAM-1 signaling caused a small increase in the instantaneous velocity of STVs (Figure 6D; SynCAM = 0.235 ± 0.021 μm/s, n = 52 movements; no SynCAM = 0.183 ± 0.012 μm/s, n = 58 movements, *P* = 0.030). Since the time-course of presynaptic protein recruitment to SynCAM-1 adhesion sites has not been established, we also imaged STVs at 24 hours after contact with SynCAM-1. After 24 hours, SynCAM-1-expressing cells had no effect on STV movement (Figure 6E; pause duration: SynCAM = 140.3 ± 15.6 seconds, n = 62 pauses; no SynCAM = 116.5 ± 14.4 seconds, *P* = 0.197, n = 71 pauses, instantaneous velocity: SynCAM = 0.202 ± 0.012 μm/s, n = 71 movements; no SynCAM = 0.171 ± 0.007 μm/s, *P* = 0.060, n = 86 movements).

Neuroligin and SynCAM interact with different presynaptic adhesion molecules [1,2,62]. Therefore, it might be expected that the effects of neuroligin and SynCAM would be additive or synergistic. However, contact with HEK293 cells that expressed both neuroligin-1 and SynCAM-1 had no effect on the movement of STVs (Figure 6F; pause duration: neuroligin/SynCAM = 124.6 ± 12.7 seconds, n = 61 pauses; no neuroligin/SynCAM = 116.7 ± 14.3 seconds, *P* = 0.482, n = 51 pauses; instantaneous velocity: neuroligin/SynCAM = 0.196 ± 0.012 μm/s, n = 63 movements; no neuroligin/SynCAM = 0.175 ± 0.008 μm/s, *P* = 0.187, n = 73 movements).

In addition to comparing the transport of STVs and PTVs in axonal regions that contact HEK293 that either did or did not express synaptogenic adhesion molecules, we also compared axonal transport inside and outside of contact regions within the same axon. Each combination of transport vesicle and non-neuronal cell treatment described above was tested. No significant (all *P* > 0.05) difference in average pause duration or instantaneous velocity was observed when comparing transport vesicles inside of contact regions versus those outside of contact regions (data not shown), with one exception. The average pause duration of STVs was significantly increased inside axonal regions that contacted SynCAM-expressing cells for 24 hours (outside contact = 105.6 ± 6.1 seconds, n = 339 pauses; inside contact = 140.3 ± 15.6 seconds, n = 62 pauses).

Together, our data suggest that while trans-synaptic signaling might have some effect on STV movement, regulation of transport is not the main mechanism through which trans-synaptic adhesion mediates synaptic protein recruitment. It is unclear whether the distinct effects of neuroligin and SynCAM on STV trafficking represent mechanistic differences in how recruitment of STVs occurs down-stream of presynaptic neurexin and SynCAM. Since neuroligin plus SynCAM did not result in the same changes as either neuroligin or SynCAM, it is possible that either (i) the observed changes are not essential for STV recruitment, or (ii) when accumulated at the same sites, presynaptic neurexin and SynCAM compete with each other for some factor that is necessary for the observed effects on STV trafficking, essentially dampening their distinct effects.

### Synaptic vesicle and active zone protein transport vesicles are not attracted to sites of trans-synaptic signaling

**Figure 7:**
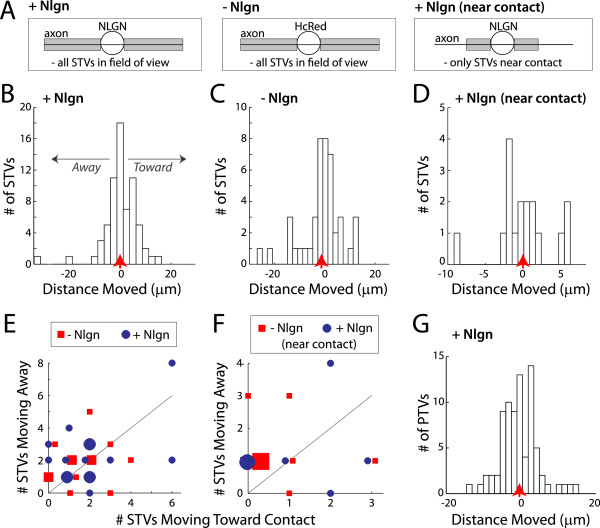
**Trans-synaptic signaling does not attract synaptic vesicle or active zone proteins. (A)** Schematics of HEK293 cell contact with an axon. Analysis was done on all STVs with initial positions either outside the contact area (*gray area, left, center*) or directly adjacent to the contact region (*gray area, right*). **(B-D)** Histograms of the net movement of STVs towards (positive) or away from (negative) contacts with HEK293 cells expressing either neuroligin-1 + HcRed (B, D) or HcRed only (C). STV movement was not biased towards sites of neurexin-neuroligin signaling, for either all STVs or only STVs adjacent to the contact. **(E, F)** Scatter plot of the number of STVs moving towards versus away from contacts with cells expressing neuroligin-1 + HcRed (*blue circles*) or HcRed only (*red squares*). Each small point represents the ratio for one axon, with larger points representing additional axons with the identical ratio. These ratios were approximately the same for axons that contact cells expressing neuroligin-1 or HcRed (*black line* = unity ratio line). **(G)** Similar to STVs, PTVs were not attracted to sites of neuroligin-1 contact. (B-D, G) *Red arrows* = mean.

Synaptogenic adhesion could recruit presynaptic machinery by actively attracting synaptic proteins to sites of trans-synaptic signaling. To test this hypothesis, we determined whether STV movements are biased towards sites of neuroligin-1 contact (Figure 7). First, we quantified the net distance and direction moved for individual STVs. Movements toward the contact were assigned positive values, while movements away from the contact were negative. This analysis indicated that neuroligin-1 signaling did not bias net STV movement toward neuroligin-1 (Figure 7B; net movement = −0.078 ± 0.857 μm, n = 67 STVs), similar to contacts with HcRed-expressing HEK293 cells (Figure 7C; net movement = −1.39 ± 1.22 μm, n = 44 STVs; *P* = 0.60). Since trans-synaptic signaling might only attract vesicles near the contact, we then restricted the analysis to STVs with initial positions adjacent to the contact, within a region equal to the contact in length. Again, neuroligin-neurexin signaling did not bias STV transport (Figure 7D; neuroligin (Nlgn) = 0.000 ± 0.888 μm, n = 17 STVs; HcRed = −3.08 ± 1.69 μm, n = 19 STVs; *P* = 0.23). Moreover, the distributions of the net movements were not altered by neuroligin-neurexin signaling (*P* > 0.15, Kolmogorov-Smirnov). In addition, neuroligin-1 did not change the fraction of STVs moving toward contacts (Figure 7E; # STVs moving toward/# STVs moving away: Nlgn = 0.89, n = 66 STVs; HcRed = 0.83, n = 44 STVs). Similarly, STVs adjacent to contacts were not attracted to contacts (Figure 7F; Nlgn = 0.89, n = 17 STVs; HcRed = 0.46, n = 19 STVs).

STVs also displayed no bias in movement towards HEK293 cells expressing SynCAM-1- or SynCAM-1 + neuroligin-1. SynCAM-1did not alter the net distance and direction moved by all imaged STVs (*P* = 0.33, n = 136 and 88 STVs for SynCAM and HcRed, respectively) or STVs adjacent to contacts (*P* = 0.23, n = 39 and 28 STVs for SynCAM and HcRed). The same was true when contacts expressed both SynCAM-1 and neuroligin-1 (all STVs: *P* = 0.70, n = 136 and 69 STVs for SynCAM + neuroligin and HcRed, respectively; near contacts: *P* = 0.55, n = 39 and 18 STVs for SynCAM + neuroligin and HcRed, respectively). Likewise, STV movement was not biased toward contacts with SynCAM-1 after 24 hours of contact (*P* = 0.13, n = 39 and 36 STVs for SynCAM and HcRed, respectively). Again, the data were not significantly different when the distributions were compared using the Kolmogorov-Smirnov test (*P* > 0.11). Furthermore, the ratio of STVs moving toward vs. away from contact regions (for SynCAM, SynCAM + neuroligin, or 24 hours SynCAM) was less than or equal to 1.13, indicating a lack of attraction to sites of trans-synaptic adhesion. Taken together, the above data demonstrate that trans-synaptic signaling does not actively recruit STVs from outside regions of contact.

Although STVs were not actively attracted to contact sites, it remained possible that trans-synaptic signaling actively recruits active zone precursors to sites of contact, followed by passive capturing of STVs. To test this, we determined whether movements of PTVs were biased toward trans-synaptic adhesion sites. Similar to STVs, PTVs showed no bias in movement towards neuroligin-1 contact sites (Figure 7G), both when including all PTVs in the analysis (Nlgn = −0.35 ± 0.56 μm, n = 79 PTVs; HcRed = 1.38 ± 0.68 μm, n = 69 PTVs; *P* = 0.071) and when limiting the analysis to PTVs with initial positions adjacent to contacts (Nlgn = −1.34 ± 1.12 μm, n = 26 PTVs; HcRed = 0.72 ± 0.86 μm, n = 28 PTVs; *P* = 0.046). When the distributions were compared using the Kolmogorov-Smirnov test, no differences were seen (*P* > 0.084). Collectively, our data indicate that trans-synaptic adhesion signaling does not attract synaptic vesicle or active zone matrix proteins to developing synapses.

## Discussion

### Presynaptic proteins are trapped at, but not attracted to, developing synapses

**Figure 8:**
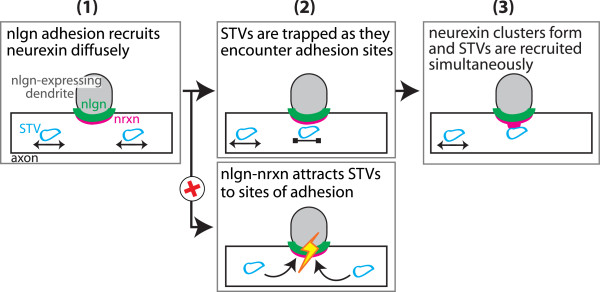
**Model of synaptic vesicle protein transport vesicle (STV) recruitment to a developing presynaptic terminal. (1)** Axo-dendritic contact induces diffuse neurexin recruitment to the contact site. **(2)** STVs are not attracted to this site (*bottom*). Instead, they are trapped as they encounter this site during normal axonal transport (*top*). **(3)** As STVs become trapped, neurexin simultaneously clusters, resulting in the formation of a stable site of synaptic protein accumulation.

Presynaptic terminal formation is initiated by contact between axons and dendrites, which leads to interactions between trans-synaptic adhesion molecules, recruitment of presynaptic proteins and subsequent organization of presynaptic terminals (Figure 8). It remains unclear how trans-synaptic signaling recruits presynaptic proteins. On one hand, trans-synaptic adhesion could actively attract transport vesicles to sites of axo-dendritic contact. Alternatively, trans-synaptic adhesion could establish sites in the axon where synaptic proteins become trapped and organized into a presynaptic terminal, without actively attracting transport vesicles.Our results favor the ‘trapping’ model (Figure 8, step 2, top). STVs and PTVs did not preferentially move towards sites of trans-synaptic signaling, and their transport was minimally altered by trans-synaptic adhesion. These findings suggest that trans-synaptic adhesion does not actively attract transport vesicles into developing presynaptic terminals. Instead, these data support the hypothesis that trans-synaptic adhesion establishes sites in the axon that capture and stabilize available presynaptic protein. Importantly, STVs were not attracted to SynCAM-1 or both neuroligin-1 and SynCAM-1, and STV movement was unaffected by this combination of trans-synaptic signaling. Therefore, assembly of synaptic proteins at developing presynaptic terminals via establishment of sites of stabilization (rather than active attraction) appears to represent a general mechanism that is conserved across synaptogenic pathways.

SV proteins preferentially accumulate at predefined sites that are intrinsic to the axon [38]. Although it is not known what stabilizes synaptic proteins at these specific sites, STVs frequently pause at these sites prior to synaptogenesis. Trans-synaptic adhesion might facilitate recruitment by stabilizing paused STVs [1,2,38]. It is important to note that STVs carry a variety of synaptic vesicle and some active zone proteins [33], so it remains unclear which proteins mediate any interactions (direct or indirect) between STVs and neurexin or SynCAM. ARL-8, which is transported with STVs in *C. elegans,* has recently been shown to control aggregation and delivery of SV and AZ proteins to appropriate sites of synaptogenesis [60,63]. Synaptogenic adhesion could act through regulation of ARL-8 or a similar pathway to stabilize paused STVs.

The trapping model predicts that the supply of synaptic proteins in the axon would determine the probability that synaptic proteins encounter these sites and, therefore, levels of trapping and recruitment. Consistent with this, synapse formation is enhanced by increasing the number of mobile STVs in the axon [64,65], and synaptic protein recruitment to poly-D-lysine coated beads preferentially occurs in axons with high levels of protein expression and mobility [66]. Interestingly, disruptions in transport have been linked to defects in synapse formation and function [60,63,67-70] and associated with intellectual disability in humans [71].

### Synaptic proteins are labile at developing synapses

While there have been studies of the stability of new synapses [12,72], it has remained unclear how stable synaptic proteins are at synapses after their initial recruitment. Here, we showed that enrichment of SV proteins at individual trans-synaptic signaling sites is highly labile: although SV proteins remained enriched at these sites, the degree of enrichment rapidly increased and decreased, oftentimes varying over the course of minutes. Similar fluctuations in synaptic protein levels have been observed at mature synapses, where changes in levels of synaptic vesicles and active zone proteins have been directly linked to changes in synapse function [73-75]. At mature synapses, lability in presynaptic protein levels arises through sharing of presynaptic components between adjacent synapses [18-21,23-25,76,77], and individual shared SVs can be functionally integrated into the synapse within minutes after their arrival [23,77]. Our results suggest that this dynamic arrival and departure (fluctuation) of SVs at synapses occurs at the earliest stages in synapse development. It remains to be seen whether SVs at developing synapses can rapidly become incorporated into the synapse and fuse with the plasma membrane in response to action potentials. However, STVs can fuse with the plasma membrane upon neuronal depolarization [27,29,38] and most likely release glutamate at these sites when they do [38]. Since glutamatergic signaling regulates the accumulation of presynaptic proteins at developing synapses [78-80], varying the level of presynaptic proteins at new excitatory synapses could alter glutamate release and contribute to feedback or auto-regulation of synapse maturation.

We observed both gradual and sudden dramatic changes in the amount of synaptic proteins recruited. These two types of fluctuations in recruitment may represent distinct features of presynaptic growth and stabilization. The sudden appearance of synaptophysin at the site of trans-synaptic adhesion indicates that presynaptic proteins can be recruited and stabilized at contacts in the order of seconds. This is striking considering recent evidence that dendritic spines can form in seconds in response to glutamate uncaging [81]. Together, these data suggest that complete synapses can form extremely rapidly. In addition, the immediate loss of synaptophysin puncta that we observed suggests that developing synapses can be eliminated equally quickly. Finally, the gradual accumulation or loss of synaptophysin that occurred at many contacts may correspond to changes in presynaptic maturation or strength [82].

### A model of neuroligin-neurexin mediated presynaptic protein recruitment

It has been proposed that synaptogenesis occurs in a two-step model: postsynaptic clusters of neuroligin induce neurexin clustering, then clustered neurexin seeds the recruitment of presynaptic proteins [16]. Our data support the hypothesis that neurexin clustering is critical for stable synaptic protein recruitment. However, we also found that neurexin clustering and synaptophysin recruitment appeared simultaneously. Although the two steps of the model could occur sequentially but very rapidly, an intriguing alternative explanation is that a cue within the axon cooperatively contributes to both neurexin clustering and STV accumulation. Consistent with this idea, recent work in *Drosophila* showed that neurexin clustering is mediated by axonal syd-1, and that this process is critical for the synaptogenic ability of neurexin [49]. Syd-1 acts upstream of syd-2/liprin-α, a protein linked to transport of STVs and initiation of active zone formation [83-89]. It will be important in the future to determine whether similar molecular mechanisms link neurexin clustering to STV stabilization in mammals. Although mammalian orthologs of syd-1 do not contain the requisite PDZ for neurexin organization, mammalian syd-1 (mSYD1A) has recently been shown to regulate SV protein accumulation during synaptogenesis and to interact with liprin-α2 [90]. Interestingly, in *Drosophila*, feedback from the postsynaptic partner also contributes to presynaptic assembly and stabilization [49]. We found that the presence of the postsynaptic scaffolding molecule S-SCAM in neuroligin-1-expressing HEK-293 cells increased the degree of neurexin clustering in the axon, consistent with analogous cooperative pre-and postsynaptic mechanisms existing in mammals.

Here, we have focused on synapse assembly down-stream of neuroligin-neurexin signaling, the prototypical synaptogenic adhesion pair. Additional factors also play a role in regulating protein levels at presynaptic terminals, including local actin polymerization [7,91-93], signaling through BDNF/TrkB [65,94-97] and NMDA receptors [80], altering VGLUT1 expression [78,79], and through other trans-synaptic adhesion molecules not addressed in our study [2]. These signals could regulate neurexin clustering, function down-stream of neurexin clustering, or operate through parallel mechanisms. Either way, these mechanisms likely work in concert to dynamically regulate synapse development and function.

## Conclusions

Our results strongly support a model of synapse formation where presynaptic proteins are trapped at developing presynaptic sites, rather than actively attracted to them (Figure 8, steps 1 to 2). Trapping tends to occur at sites of neurexin clustering, but not at sites of diffuse neurexin recruitment, supporting the hypothesis that the spatial arrangement of neurexin is critical for neurexin/neuroligin induced synaptogenesis. Neurexin clustering and SV protein recruitment occur simultaneously (Figure 8, step 3). Finally, at individual nascent presynaptic terminals, SV protein levels can vary within minutes to hours, and these levels correlate with the amount of clustered neurexin. These fluctuations might, in turn, affect later stages of synapse formation, maturation and function.

## Methods

All studies were conducted with an approved protocol from the Case Western Reserve University Institutional Animal Care and Use Committee, in compliance with the National Institutes of Health guidelines for the care and use of experimental animals.

### Neuronal culture and transfection

Primary neuronal cultures were prepared from rat or mouse cortices on 18 mm glass coverslips as described previously [38,41,98,99] and maintained in Neurobasal A media with B27 Supplement (Invitrogen, Carlsbad, CA, USA). At 6 to 9 days *in vitro* (DIV) and 24 to 48 hours prior to imaging, neurons were transfected using Lipofectamine 2000, essentially according to the manufacturer’s instructions (Invitrogen, Carlsbad, CA, USA). DNA was used at 1 μg per 18 mm coverslip with the exception of GFP-bassoon, which was transfected at 2 μg per 18 mm coverslip due to its large size. This amount of DNA results in efficient labeling of transport vesicles without significant over-expression within axons [33,38,41,99]. Even so, neurons were chosen for imaging that displayed moderate expression levels of the transfected constructs to avoid any possible effects of over-expression. For double transfections, the localization and movement of fluorescently-tagged proteins appeared similar to single transfections. In addition, synaptic vesicle protein transport vesicles (STVs) labeled with synaptophysin-RFP and synaptophysin-GFP were similar in size, movement, and localization.

Synaptophysin-GFP, GFP-bassoon (GFP-Bsn 95-3938), synaptophysin-mRFP, synaptophysin-mcherry, and neurexin-1β-GFP were generous gifts of Drs. Jane Sullivan (University of Washington, Seattle, WA, USA), Thomas Dresbach (Georg August University, Gottingen, Germany), Jurgen Klingauf (University of Muenster, Germany), Matthijs Verhage (Vrije Universiteit, Amsterdam, Netherlands), and Camin Dean (The European Neuroscience Institute, Gottingen, Germany). These constructs have been shown to be functional and properly localized and have been used previously for studies of protein trafficking and localization [40,54,99-102]. Fluorescent proteins attached to bassoon and synaptophysin effectively label PTVs/active zones and STVs/synaptic vesicles, respectively [31,33,34,40,41,53,54,99,100]. RFP670 was purchased from Addgene (Cambridge, MA, USA) [plasmid # 45457]. Neurexin-tdTomato was generated by seamlessly replacing the GFP sequence in the neurexin-GFP construct with tdTomato via Gibson Assembly (New England Biolabs, Ipswich, MA, USA).

### HEK293 cell culture and transfection

HEK293 cells were cultured and maintained in DMEM supplemented with 10% fetal calf serum and penicillin/streptomycin (Invitrogen, Carlsbad, CA, USA; HyClone, Logan, UT, USA). Cells were transfected 24 to 48 hours prior to dissociation and dropping onto neuronal cultures. A 3:2 or 2:1 ratio of trans-synaptic adhesion DNA to HcRed DNA (Clontech, Mountain View, CA, USA) was used to ensure co-expression of both types of DNA constructs in the same HEK293 cell [38]. To confirm co-expression, HEK293 cells were transfected with SynCAM-GFP, HA-neuroligin-1, and HcRed. After 48 hours, cells were fixed and labeled with anti-HA antibody to detect HA-neuroligin-1. Over 85% of HEK293 cells transfected with HcRed also expressed SynCAM-GFP and HA-neuroligin-1 (data not shown). HA-neuroligin-1 and HA-SynCAM-1 were generous gifts of Drs. Peter Scheiffele (University of Basel, Switzerland) and Philip Washbourne (University of Oregon, Eugene, OR, USA) and have previously been shown to recruit synaptic proteins in the hemi-synapse assay [11,103]. Myc-tagged S-SCAM was purchased from Addgene (Cambridge, MA, USA) [plasmid # 40213] [104].

### Imaging

Imaging was performed with a C1 Plus confocal system on a Nikon Eclipse Ti-E microscope utilizing a 40× Nikon Plan Apo 0.95NA objective. Lasers used for excitation were 488 nm argon and 543 nm and 633 nm helium-neon, while detection filters were 515/30 nm bandpass for GFP, 590/50 nm bandpass for HcRed/mcherry/mRFP/tdTomato, and 650 longpass for RFP670. For multi-color imaging, channels were imaged sequentially to avoid bleed-through. To avoid focal plane drift, the Perfect Focus System (Nikon, Tokyo, Japan) was used.

#### Long-term imaging

Transfected HEK293 cells were dissociated with Hank’s-based, enzyme-free cell dissociation buffer (Invitrogen, Carlsbad, CA, USA). Cells were resuspended in neuronal media then 8 × 10^4^ cells were dropped onto each coverslip of transfected neurons. Contacts between transfected axons and transfected HEK293 cells were imaged, with axons identified by their morphological characteristics [38,99]. Dual-color images were collected every 10 seconds for 7.5 minutes, with scan times for both frames not exceeding 3.3 seconds. This interval yields high temporal resolution of STV movement with minimal phototoxicity [41]. This imaging protocol was repeated every hour for the next 4 hours then every 2.5 hours for up to 25 hours. To ensure that cells remained healthy, imaging was performed in a custom made environmental chamber held at 32°C. The pH was maintained at physiological levels by equilibration in 5% CO_2_ for 15 to 30 minutes prior to sealing with vacuum grease (Dow Corning, Midland, MI, USA).

#### Short-term imaging

Synaptogenesis was induced as described above. After 1 to 4 hours, contacts between transfected axons and transfected HEK293 cells were imaged every 10 seconds for 7.5 minutes with constant perfusion of artificial cerebrospinal fluid (ACSF: 120 mM NaCl, 3 mM KCl, 2 mM CaCl_2_, 30 mM D-glucose, 20 mM HEPES, and 0.2% sorbitol, pH 7.3) at 25°C. At this temperature, STV movement is not significantly altered compared to STV movement at physiological temperatures [38]. In previous studies, the fastest velocities recorded for STV movements were approximately 1 μm/s [33,34,38], while maximal PTV velocities were lower [53,57]. Vesicles moving at this maximal velocity would move 10 μm between frames during of imaging, a small fraction of the total axonal area typically imaged. Therefore, the rapid imaging of STVs/PTVs performed here enabled reliable identification and tracking of vesicles.

#### Neurexin-GFP recruitment

Axons expressing neurexin-GFP were imaged for at least five minutes before contact with HEK293 cells to identify neurexin-GFP that was previously clustered through endogenous mechanisms. HEK293 cells transfected with HA-neuroligin-1 plus HcRed were added to the imaged coverslip until at least one transfected HEK293 cell made contact with the neurexin-expressing axons. This contact was then imaged for up to two hours at a rate of one frame/minute.

### Analysis

STVs and PTVs were manually tracked using custom written macros for ImageJ (NIH, Bethesda, MD, USA), while blind to the locations of the HEK293 cells. Analysis was restricted to axons that did not move during imaging and where at least one vesicle moved within the imaged area. To facilitate tracking, imaging was conducted on axons with an intermediate to low density of STVs or PTVs. Movements were quantified by importing the positional data into Matlab and subjecting them to custom written analysis (Mathworks, Natick, MA, USA). Pausing was defined as a period of at least 10 seconds where the velocity was lower than 0.1 μm/s, as previously described [38,41]. Vesicles that did not move during imaging were not included in this analysis, and analysis was restricted to vesicles that could be tracked throughout the imaging period. Therefore, very long pauses and vesicles that moved at high velocities for long periods of time might be underrepresented. Considering the scan speed, pixel density, and average size of transport vesicles, a typical STV or PTV was imaged within 10 to 50 ms.

Integrated density analysis was performed using custom-written macros in ImageJ that determined the location and integrated density of each transport vesicle on the axon for each image in a sequence. Contact regions were defined as axonal regions that overlapped with fluorescence from a transfected HEK293 cell. ‘% Enrichment’ was determined by the following equation:

$$\begin{array}{ll} \;\%\text{Enrichment}= & [\left({\text{IntDen}}_{\text{in}}‒{\text{IntDen}}_{\text{out}}\right)/({\text{IntDen}}_{\text{in}}\\ & +{\text{IntDen}}_{\text{out}})]*100 \end{array}$$

Where IntDen_in_ corresponded to the mean integrated density per μm of axon length inside the contact, and IntDen_out_ corresponded to the mean integrated density per μm outside the contact for the same axon. Contacts were considered to display recruitment when the% enrichment was (i) positive for at least 70% of time points 4+ hours after contact was initiated and (ii) greater than 10% for at least half of all time points 4+ hours after contact initiation. Data are shown as the mean ± standard error when applicable. Significance was determined via Wilcoxon rank sum test unless otherwise indicated. For correlation data, r = Pearson’s linear correlation coefficient and *P*-values were determined using Student’s *t*-test.

## Abbreviations

AZ: active zone; DIV: days *in vitro*; DMEM: Dulbecco’s modified Eagle’s medium; GFP: green fluorescent protein; IntDen: integrated density; Nlgn: neuroligin; PTV: Piccolo-Bassoon transport vesicle; STV: synaptic vesicle protein transport vesicle; SV: synaptic vesicle.

## Competing interests

The authors declare that they have no competing interests.

## Authors’ contributions

LB participated in the design of the study and drafted the manuscript, in addition to performing imaging experiments and analyzing data. SS participated in the design of the study and drafted the manuscript. Both authors read and approved the final manuscript.

## Supplementary Material

Additional file 1 **Example of extended imaging of a neuron-HEK293 cell contact.** An axon that was transfected with synaptophysin-GFP (*green*) and made contact with a neuroligin-expressing HEK293 cell (*magenta*) was imaged for 17.5 hours. Movie was generated by concatenating multiple short imaging sequences (1 frame per 10 seconds for 7.5 minutes) of the same contact over time. Each short sequence was separated by 1 to 2.5 hours. Time after contact was established between the neuron and HEK293 cell is indicated in the upper left-hand corner, scale bar is in the lower right-hand corner. Click here for file

Additional file 2 **Example of extended imaging of axo-dendritic contact.** An axon that was transfected with synaptophysin-GFP (*green*) and made contact with a dendrite from a different neuron that expressed RFP (*magenta*) was imaged for nine hours. Movie was generated by concatenating multiple short imaging sequences (1 frame per 10 seconds for 7.5 minutes) of the same contact over time. Each short sequence was separated by 1 to 2.5 hours. Time after imaging commenced is indicated in the upper left-hand corner; scale bar is in the lower left-hand corner. Click here for file
